# Are the Digestive Disturbances of Teething Infants, Reflexes or Not?

**Published:** 1907-07

**Authors:** W. F. Graham

**Affiliations:** Summerville, S. C.


### ARE THE DIGESTIVE DISTURBANCES OF TEETHING INFANTS, REFLEXES OR NOT?

By Dr. W. F. Graham, Summerville, S. C.

The theory of reflex nervous action generally held is that the advancing tooth exerts direct pressure on the fibrous gum tissue and that this is sufficient to set up grave constitutional disorders through reflex nervous action, some observers hold that there is also counter pressure on the nerve tissue at the incomplete root apex of the erupting tooth and that this in itself is sufficient to cause trouble.

If this reflex nervous action theory is true, why is it abolished when the child erupts the permanent teeth?

Take for example the six year molar, a large tooth with a broad surface, that breaks through gum tissue that certainly must be denser than the gum tissue of an infant, yet you cannot find in medical or dental literature any observed reflexes at this age.

The only tooth in the permanent set causing reflex nervous action is the wisdom tooth and the condition set up is always of a neuralgic character. The lower wisdom tooth is usually the offender for the gum tissue over that tooth is very dense from the fact that the superior 12-year-molar overlaps posteriorly the lower 12-year-molar and the force of mastication is partly expended on the lower gum tissue.

In the infant the digestive, absorbent, and capillary system are more completely developed than the nervous, muscular, or osseous, and there's a reason for there must be a very rapid growth of new tissue besides a repair of waste and consequently the digestive and associated organs do a comparatively larger amount of work than in the adult and that means constantly a large and free flowing blood supply to bear material to the cells.

Now in the infant as well as the adult we have in the alimentary canal at all times the putrefactive bacteria.

These saprophytic bacteria are essential to life and while innocuous within limits they can become pathogenic through excessive numbers.

Cheyne in a series of experiments on rabbits found that the common saprophyte, Proteus vulgaries was pathogenic when injected in the dorsal muscles in sufficient numbers, 225 million were required to cause death, 9 to 112 million caused a local abscess, less than 9 million produced no result. Now in some of the rabbits it took much less than 225 million bacteria to cause death and we can deduce from that fact as well as the varying quantities necessary to produce a local abscess that the individual resistance of the rabbits varied greatly but there was a limit beyond which they all succumbed.

This principle holds true with human beings for we are constantly exposed to disease germs and do not contract the disease through the fact that our individual limit of resistance has not been reached,

To the leucosytes or white blood corpuscles belongs the function among other things restraining and keeping in order the putrefactive bacteria.

Now keeping this data in mind let us investigate the condition of an infant erupting teeth.

The digestive organs are in a full blooded state and as the tooth starts to erupt nature sends a reflex to the digestive organs to get ready for harder work as the teeth are coming, that reflex causes an increased blood supply to these organs already full. At this time hygiene of the mouth is usually neglected on account of local tenderness, now should the abdominal surface become chilled we have the cutaneous blood supply driven to the deeper organs and they become congested and the white blood corpuscles are restricted in their movements and as a result of this combination of causes the putrefactive bacteria increase in overwhelming numbers and take charge of the alimentary canal and nature tries to throw them off through diarrhoeas, etc. The drug treatment indicated is laxative and not astringent.

Why are medical men who judicously prescribe calomel generally successful in the treatment of these disorders? Because calomel stimulates the liver, promotes a flow of bile and bile is nature's own intestinal antiseptic that inhibits the putrefactive bacteria.

If the infant is bottle-fed we have an additional element of danger in the introduction of putrefactive bacteria with the food as germs that breed in favorable media acquire greater virulence, and milk is an ideal culture medium.

Statistics bear out the fact that artificial fed infants are more liable to digestive disturbance and the mortality is greater among them which goes to show that other factors than the teeth must be considered.

Now these digestive disturbances have been shown to be due to a congestion of circulation in the internal organs and the multiplication of the putrefactive bacteria, and the question is what can be done to prevent this blood stasis.

By the use of an abdominal flannel band, properly applied and constantly used until the summer following the eruption of the last temporary molar you can in the majority of cases prevent any digestive disturbances, for it will keep up the capillary circulation of the abdomen and prevent any sudden chilling of the skin surface. The majority of abdominal bands fail in their purpose because they are not properly secured.

Should an infant wearing an abdominal band have any digestive trouble with the eruption of the first tooth it would show a low limit of resistance to the putrefactive bacteria and if there was no other way of accounting for the trouble I would recommend lancing the gum in order to modify the physiological reflex.

I would recommend lancing of the gums only where there was much local tenderness and inflammation.

Netter found in the salivary secretions of 15% of the healthy individuals examined, 165 in all, the micrococcus of pneumonia. Von Besser, in 81 cases examined, of nasal mucus, found dipococcus 14, S pyogenes aureses 14 times and streptococcus pyogenes 7 times; this shows the importance of mouth hygiene in the infant.

